# The beneficial impact of filtration surgery on antiviral therapy cessation in patients with cytomegalovirus-related secondary glaucoma

**DOI:** 10.1186/s12886-021-02155-3

**Published:** 2021-11-08

**Authors:** Yusuke Murai, Sotaro Mori, Fumio Takano, Kaori Ueda, Mari Sakamoto, Takuji Kurimoto, Sentaro Kusuhara, Yuko Yamada-Nakanishi, Makoto Nakamura

**Affiliations:** Department of Surgery, Division of Ophthalmology, Kobe University Graduate School of Medicine, 7-5-1 Kusunoki-cho, Chuo-ku, Kobe, 650-0017 Japan

**Keywords:** Antiviral therapy, Cytomegalovirus-related keratouveitis, glaucoma surgery, Trabeculectomy

## Abstract

**Purpose:**

Cytomegalovirus (CMV)-related keratouveitis elevates intraocular pressure (IOP). Antiviral therapy does not always control IOP and some patients do not tolerate systemic antiviral therapy because of the side effects. The purpose of this study is to evaluate the clinical characteristics of patients with CMV-related keratouveitis and determine the impact of glaucoma surgeries on the postoperative antiviral therapy regimen.

**Methods:**

We enrolled twenty-two patients with CMV-DNA-positive keratouveitis between June 2012 and July 2019 in Kobe University Hospital. The following clinical parameters were collected: gender, age, history of previous intraocular surgery, antiviral medications, visual acuity, IOP, glaucoma drug score, corneal endothelial cells density, and the mean deviation of a Humphrey visual field test at the first visit and before and 1 year after glaucoma surgery.

**Results:**

All twenty-two patients started on oral and/or topical antiviral therapy. Eighteen patients needed glaucoma surgery despite their antiviral medications. Nine patients underwent trabeculotomy (TLO) and nine underwent trabeculectomy (TLE) as the first surgical intervention. Six of patients who initially underwent TLO and two of the patients who initially underwent TLE required additional TLE within 1 year. Each of the 15 patients who underwent at least 1 TLE showed a reduction in the magnitude and variation of IOP and glaucoma drug scores and 13 patients were able to discontinue antiviral therapy. For the remaining 4 patients, IOP and inflammation were controlled but with antiviral medications.

**Conclusions:**

In patients with CMV-related keratouveitis, TLE decreases and stabilizes IOP and contributes to withdrawal from antiviral medications.

## Background

Cytomegalovirus(CMV) causes iridocyclitis [1], elevated intraocular pressure (IOP) [2], and corneal endothelitis [3, 4] in immunocompetent patients. It is also a known cause of Posner-Schlossman syndrome (PSS) [2, 5–8] and Fuchs’ heterochromic iridocyclitis (FHI) [2]. Although topical steroid and ocular hypotensive eyedrops are initiated for the treatment of iridocyclitis and elevated IOP, respectively, the control of inflammation and ocular hypertension in many patients requires antiviral treatments, which include intravenous, oral, topical, or intravitreal ganciclovir administration [6, 9–15]. However, these combinations of medical therapy fail to reduce IOP in some cases, necessitating glaucoma surgery [8, 16, 17].

There are few reports that evaluate whether trabeculotomy (TLO) is effective for CMV-related glaucoma, given the potential for steroid-induced ocular hypertension, or whether a filtering surgery like trabeculectomy (TLE) is a more-suitable surgical intervention [18]. Moreover, although long-term systemic antiviral therapy sometimes causes pancytopenia or liver dysfunction, withdrawing antiviral medications has been reported to increase the risk of inflammation recurrence [6, 10, 14, 19]. Thus, there is still in controversy around whether or not these costly antiviral therapies should continue even after glaucoma surgery.

This study evaluated the clinical characteristics of patients with CMV-associated keratouveitis and elevated IOP, with a particular focus on the impact of glaucoma surgery on the discontinuation of antiviral therapy.

## Methods

### Patients

We retrospectively reviewed the clinical records of patients with CMV-related keratouveitis who were treated at Kobe University Hospital between June 2012 and July 2019. The study adhered to the tenets of the Declaration of Helsinki and was approved by the Institutional Review Board of Kobe University (No. 15571). Informed consent was not obtained from each patient because this was a retrospective, observational study. However, patients were able to withdraw consent anytime by giving information about this study opened on the hospital homepage as an opt-out choice.

None of these patients presented with CMV retinitis. These patients had presented with a unilateral IOP spike with minimal anterior chamber inflammation and keratoprecipitation simulating PSS, corneal endothelitis with a decrease of corneal endothelial cell density (ECD), or anterior uveitis with iris hypopigmentation, such as FHI. An aqueous tap was performed to detect CMV-DNA and the assays were outsourced to a major commercial laboratory (SRL Inc., Tokyo, Japan). The diagnosis of CMV-related keratouveitis was eventually determined by the detection of CMV-DNA by polymerase chain reaction (PCR) in the aqueous humor, with more than 1.0 × 10^2^ copies/mL of CMV-DNA considered significant.

### Ocular biometrics

The following clinical parameters were collected for each patient: gender, age, location of pathology (left or right eye), history of previous intraocular surgery and follow up, antiviral medications, visual acuity, IOP, glaucoma drug score, ECD, and the mean deviation of a Humphrey visual field test (HVF) at the first visit and before and 1 year after glaucoma surgery. The IOP was measured twice per session using a Goldmann applanation tonometer and the average value was recorded. When the difference between the two measurements exceeded 1 mmHg, a third measurement was carried out and the median value of the three measurements was designated as the IOP of the session. Best-corrected decimal visual acuity (VA) was measured using a Landolt ring chart and was converted to the logarithm of minimal angle resolution (logMAR) for statistical analyses. In this study, VA of 0.01 and counting finger denoted a logMAR of 2.0, and hand motion was scored as 2.9 [20]. There were no patients whose VA was light perception or loss of light perception. Noncontact-type specular microscopy (Noncon Robo SP-8000; Konan Medical, Tokyo, Japan) was used to measure ECD in the central area of the cornea. We used the Swedish interactive threshold algorithm standard 30-2 program of the HVF analyzer (Carl Zeiss-Meditec) to measure the visual field.

### Antiviral therapy

In our hospital, we used orally administered valganciclovir (Mitsubishi Tanabe Pharma; Osaka, Japan) and ganciclovir eyedrops as antiviral therapy for CMV-related keratouveitis. Valganciclovir 450 mg was administrated orally twice a day for 21 days as an induction regimen in patients with normal renal function. This was followed with a maintenance dose of 450 mg once daily. Ganciclovir eyedrops with a concentration of 0.5% was house-made in a dispensing facility of the Kobe University Hospital by diluting 500 mg intravenous ganciclovir substance (Mitsubishi Tanabe Pharma) with physiological saline. The patients started to receive eyedrops four times a day, which was tapered and terminated, if possible, according to the degree of intraocular inflammation and IOP control. TLO or TLE with intraoperative 3-min 0.04% mitomycin-C (Kyowa Kirin, Tokyo, Japan) was performed when IOP could not be controlled even with the combinations of topical ocular hypotensives and steroids together with the aforementioned antiviral therapy.

### Statistical analyses

In this study, all quantitative variables did not follow a normal distribution with the Shapiro-Wilk test. Thus, we denoted these data as the median and interquartile range. A comparison of the parameters between the values preoperatively and 1 year after surgery was carried out using the Wilcoxon signed-rank test. Correlations of logMAR, ECD, and HVF median deviation between the first and last visits were tested using Spearman’s rank correlation coefficients. Statistical analysis was performed using Medcalc (version 19.5.1, Medcalc software, Mariakerte, Belgium) and *P* < 0.05 was considered statistically significant.

## Results

Table 1 shows the demographic characteristics of 22 patients in whom CMV-DNA was detected in the aqueous humor. Nineteen (86%) of 22 patients were male. The median duration from onset to referral to our hospital was 6.5 years. Almost all patients presented uncontrolled IOP elevation even though they had maximally tolerated glaucoma medications and steroid eyedrops. Thus, the median IOP and glaucoma drug score were as high as 32 mmHg and 4, respectively, at their first visit to our hospital. The ECD could not be measured in one patient and the HVF could not be measured in three patients at their first visit because their glaucoma stages were too advanced.

**Table 1:** Clinical parameters of 22 patients collected at the first visit

	Number	%	
Men	19	86	
Right eye	10	45	
	Number	Median	Interquartile range
Age	22	68.5	65.3–72.5
Age at first appearance of symptoms, Yrs	22	65	51.3–68.8
Duration from onset to referral to our hospital, Yrs	22	6.5	3.3–10
CMV PCR, copies/mL	22	2.1 × 10^3^	6.8 × 10^2^–6.2 × 10^4^
LogMAR	22	0	−0.08–0.15
Intraocular pressure, mmHg	22	32	20.8–42
Glaucoma drug score	22	4	3.3–4
Corneal endothelial cell density, cells/mm^2^	21	2188	1314–2674
HVF mean deviation, decibel	19	−4.63	−2.04–15.36

*CMV* Cytomegalovirus, *PCR* Polymerase chain reaction, *HVF* Humphrey visual field test

**Fig. 1:**
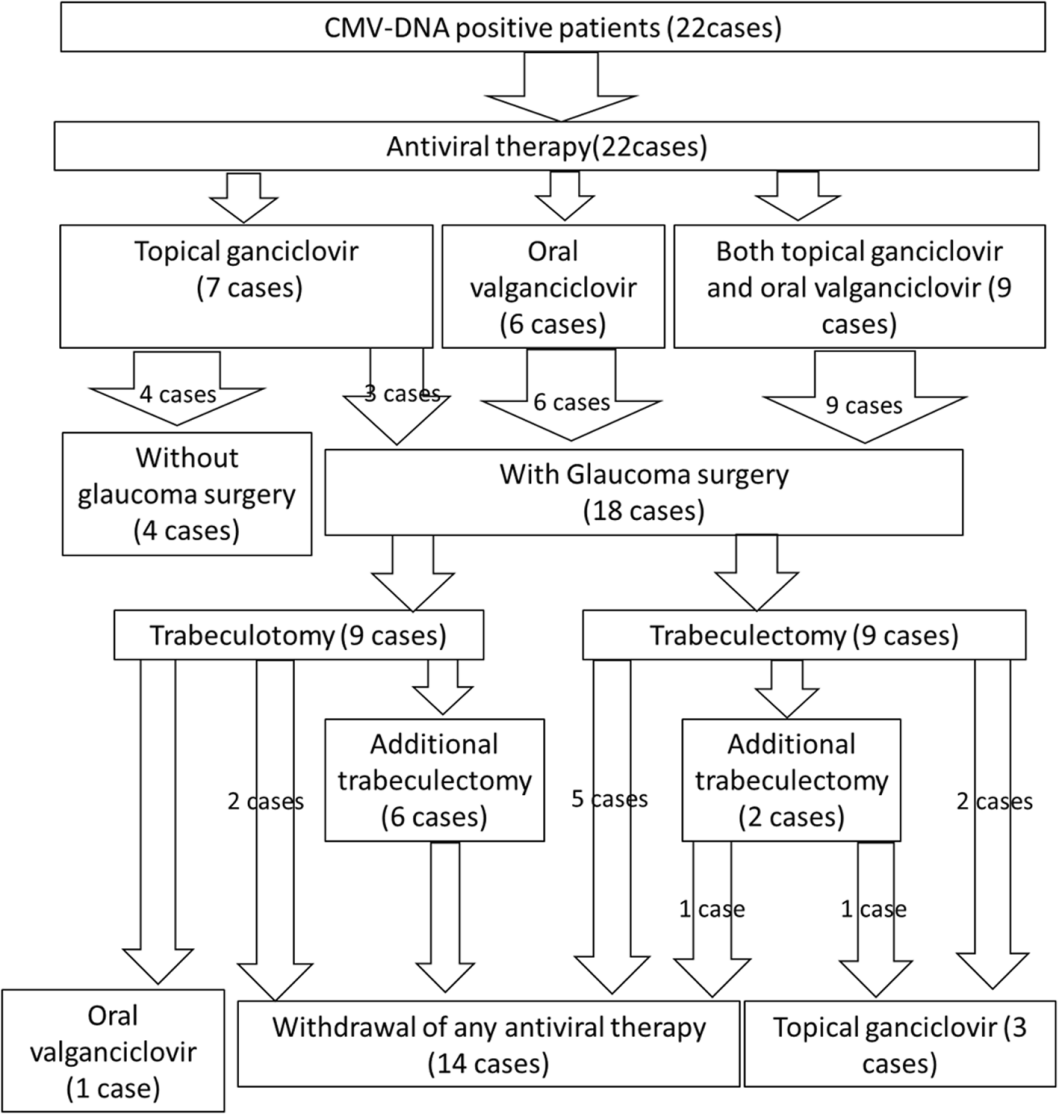
Flowchart outlining the authors’ process of care for patients positive for CMV-DNA

Figure 1 summarizes the treatment flows of patients in this study. A total of 22 eyes in 22 patients were enrolled as the CMV-related anterior uveitis. Four patients presented elevated IOP, iridocyclitis, and corneal endothelitis, but the elevated IOPs were controlled by glaucoma and ganciclovir eyedrops without a glaucoma surgery.

The remaining 18 patients needed glaucoma surgery. Nine out of these 18 patients underwent TLO as the initial glaucoma surgery, of whom six (67%) required additional TLE within 1 year due to uncontrolled IOP. Of the remaining three patients who underwent TLO but did not undergo additional TLE, one required continuous oral administration of valganciclovir and two could withdraw from antiviral therapy by 1 year after surgery. The remaining 9 patients underwent TLE as the initial glaucoma surgery, of whom two required a second TLE. Thus, a total of 15 patients received at least 1 TLE. Each of the six patients who underwent an additional TLE following initial TLO could discontinue the antiviral medications. Among the nine patients who had TLE as their initial glaucoma surgery, five patients without an additional TLE and one patient with an additional TLE could withdraw from their antiviral therapy, while the remaining 3 patients needed to continue the topical ganciclovir.

**Fig. 2:**
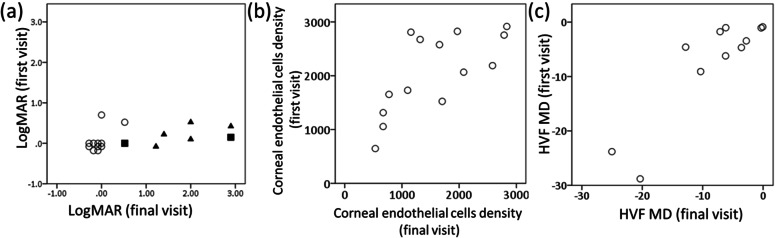
Scatter plots for (**a**) logMAR, (**b**) corneal endothelial cells density, and (**c**) Humphrey visual field test mean deviation between first and final visit. There was a significant correlation between the first and final visit of all parameters (Spearman’s rank correlation coefficient (**a**) *P* = 0.008 (**b**) *P* = 0.003 (**c**) *P* = 0.001). **a** Squares indicate patients whose visual acuity (VA) decreased due to cornea decompensation, whereas triangles indicate patients whose VA decreased due to glaucoma progression. HVF, Humphrey visual field test; MD, mean deviation

Figure 2 depicts scatter plots of the logMAR, ECD, and HVF median deviation in all 22 cases at the last visit as compared with the first visit to our hospital. The median (interquartile range) follow-up period was 3.9 (2.7, 6.9) years. There was a significant correlation of all three parameters between the first and last visit (*P* < 0.01, Spearman’s rank correlation coefficient). During follow up, ECD could not be measured in three patients and visual field examination could not be conducted on the HVF in five patients.

Table 2 summarizes preoperative and 1-year postoperative ophthalmic parameters of the 15 patients who underwent at least 1 TLE. Values after a second TLE were adapted in patients who received TLE twice. The data suggest that TLE significantly decreased the magnitude of IOP, IOP standard deviations, and glaucoma drug scores (all parameters had a significance of *P* = 0.0001, Wilcoxon signed-rank sum test). The ECD in two patients in whom the preoperative value was less than 1000/mm^2^ could not be measured postoperatively.

**Table 2:** Outcomes of 15 patients managed with trabeculectomy

	Preoperative	Number	1 year after last surgery	Number	*P* value
LogMAR	0 (−0.08. 0.31)	15	0 (−0.13, 0.82)	15	0.01
IOP, mmHg	29 (24, 31)	15	11.5 (8, 13.3)	15	0.0001
IOP standard deviation, mmHg	8.7 (7.0, 12.0)	15	3.6 (1.8, 4.4)	15	0.0001
Glaucoma drug score	5 (4, 6)	15	0 (0, 1)	15	0.0001
Corneal endothelial cell density, cells/mm2	1573.5 (1197, 2098.8)	14	1349.5 (1051, 2151)	12	0.42
HVF mean deviation, decibel	−11.08 (−3.21, −21.08)	12	−8.28 (−2.77, −18.01)	8	0.54

Values are expressed as median (lower, upper quartile value). A comparison between the two groups was carried out using Wilcoxon signed-rank sum test. *IOP* Intraocular pressure, *HVF* Humphrey visual field test

Table 3 summarizes the four patients who did not need glaucoma surgery. Patient 1 initially presented with the uncontrolled IOP elevation and iritis. An antiviral therapy was started immediately after the presence of CMV-DNA was confirmed in the aqueous humor. The medication decreased the IOP to a normal range, allowing a withdrawal of glaucoma eyedrops. As of the last visit 4.8 years after the first visit, the patient took only ganciclovir eyedrops and showed good IOP and inflammation control even without oral valganciclovir. Patients 2, 3, and 4 had iritis and corneal endothelitis and the IOP was controlled by topical therapy without surgery. Patient 2 initially received, and continue to use, ganciclovir eyedrops without oral antiviral therapy. Patients 3 and 4 initially received antiviral therapy for iridocyclitis, but could withdraw both oral and topical antiviral medications at their last visit after 3.6 and 3.2 years, respectively, from their discontinuance of antiviral therapy.

**Table 3:** Clinical parameters of four patients treated without surgery

First visit							Last visit					
Sex	Age at first visit, Yrs	Duration from onset to referral to our hospital, Yrs	CMV PCR, copies/mL	LogMAR	IOP, mmHg	ECD, cells/mm^2^	Follow-up period, Yrs	LogMAR	IOP, mmHg	ECD, cells/mm^2^	Oral valganciclovir	Topical ganciclovir
F	33	8	1.2 × 10^4^	−0.08	33	2457	4.8	−0.18	13	2075	No	Yes
M	83	6	1.1 × 10^2^	0.10	14	449	1.9	2.9	18	unmeasurable	No	Yes
M	72	18	4.2 × 10^3^	0.15	16	2463	5.8	0	10	1855	No	No
M	68	7	8.8 × 10^4^	−0.18	17	2410	4.2	−0.18	16	1792	No	No

*CMV* Cytomegalovirus, *PCR* Polymerase chain reaction, *IOP* Intraocular pressure, *ECD* Corneal endothelial cells density

## Discussion

The present study demonstrates that TLE is a powerful intervention to manage IOP control, suggesting that TLE increased the chance to withdraw antiviral medications. Trabeculotomy was a less-effective intervention in this study.

At the last visit, five patients underwent severely reduced VA owing to damage to the central visual field induced by glaucoma progression. Traditionally, patients with PSS are less likely to develop visual field defect. However, because it has been known that the clinical characteristics of PSS are simulated in a fraction of patients with CMV-related anterior uveitis [2, 5, 21], clinicians should be aware of the presence of patients who clinically present with PSS but quickly develop a visual field defect with CMV-related keratouveitis. Shirahama et al. [17]. reported that patients with CMV-associated anterior uveitis are at a higher risk for, and have a faster progression of, glaucoma.

In our cohort with a history of elevated IOP due to CMV-related keratouveitis, 10 (46%) patients had no or mild visual field defects with a mean deviation <− 8 dB, 2 (9%) patients had a moderate stage of glaucoma between − 8 and − 20 dB, and 10 (45%) patients had glaucoma of an advanced stage with either a mean deviation > − 20 dB or intolerance of HVF due to the loss of the central vision during follow up. Patients in this cohort who demonstrated CMV-related secondary ocular hypertension were polarized into those with mild and those with advanced stages of glaucomatous damage. This suggests that the progression of visual field damage may be rapid when developed in patients with CMV-related keratouveitis and elevated IOP and that early intervention of IOP control is critical to maintain visual field and quality of vision.

Previous reports showed that CMV infects the trabecular meshwork cells and reorganizes actin cytoskeleton, increasing aqueous humor outflow resistance [22, 23]. Given the similar mechanism, we expected TLO to show good surgical outcome in CMV-related secondary glaucoma as in steroid-induced glaucoma [24]. However, the present result was disappointing in that 6 of 9 patients who had TLO as the initial glaucoma surgery required additional TLE less than 1 year postoperatively. We speculated that in patients with CMV-related glaucoma, both the trabecular meshwork cells and the post-meshwork aqueous outflow pathway were impaired, leading to a failure of the reconstruction of the physiological aqueous humor outflow facility through TLO. In three patients who had good IOP control after TLO, the mechanism of steroid-induced ocular hypertension rather than the CMV-induced destruction of conventional outflow may have been implicated. Compared with TLO, TLE showed an excellent result in this study, which was consistent with the results in other previous reports [7, 21, 23, 25].

Several reports indicated that the number of CMV viral loads in the anterior chamber is associated with IOP elevation and ECD reduction [26, 27]. Trabeculectomy may drain virus loads out of the anterior chamber as a nature of filtration surgery, stabilizing IOP and preventing ECD loss [7, 23, 25]. Our data actually demonstrated a significant decrease in the IOP standard deviation after TLE. Taken together, TLE is a good procedure to prevent the postoperative IOP spike induced by CMV-related keratouveitis.

An additional finding of the present study is that TLE could increase the chance to withdraw from the antiviral medications. All of 15 patients who had TLE received antiviral therapy preoperatively. All of the nine patients who received oral valganciclovir preoperatively terminated it after TLE, while 8 of 11 patients who received topical ganciclovir eyedrops discontinued its use during the median 3.0-year postoperative follow-up period. Although effective, antiviral therapy is deemed to have a weak point of recurrence after discontinuation in CMV-related keratouveitis. De Schryver et al. [19] reported 3 (60%) of five patients showed recurrences after termination of intravenous injection. Chee et al. [2] reported a recurrence rate of 70% (7 of 10 patients), while Touhami et al. [14] reported a recurrence rate of 69% (9 of 13 patients) when ganciclovir was orally administered and 76% (16 of 21 patients) when injected intravenously.

Compared with these studies, our study tended to have a higher rate of withdrawal from the antiviral medications after TLE and we hypothesized two reasons for this. One hypothesis is a possible suppression of the recurrence of CMV-associated iridocyclitis per se by filtration surgery. Second one is that the symptomatic events of IOP elevation and iridocyclitis are reduced due to aqueous filtration even though the recurrence rate remains unchanged.

We believe that an antiviral therapy is an initial option to treat CMV-associated secondary glaucoma when topical steroid or glaucoma eyedrops could not work. We also recommend considering glaucoma surgery, especially filtering surgery, when patients do not tolerate antiviral therapy due to systemic side effects or when discontinuation of antiviral therapy induces recurrences. Given that patients with the clinical characteristics of PSS show null or mild glaucoma visual field defects even with IOP spikes, TLO rather than TLE may be more likely selected for such patients. However, we should be aware that the effectiveness of TLO is limited for patients with CMV-associated secondary glaucoma. When we compared the CMV-DNA copy number among seven patients who did not need TLE and 15 patients who needed TLE, we found no significant difference (*P* = 0.75, Mann-Whitney U test). A previous report demonstrated that the virus load of CMV did not correlate with the severity of ocular inflammation, unlike herpes simplex virus and varicella-zoster virus [28].

The current study has two limitations. We did not evaluate the CMV-DNA number after TLE; thus, we could not show direct evidence that TLE could suppress CMV proliferation in the anterior chamber. In addition, we could not know whether TLE might prevent decreasing ECD due to CMV-related anterior uveitis, because we did not measure ECD over time after surgery. A long-term prospective study is required to address this issue. In this study, we could not measure the anterior chamber inflammation by flare meter. The flare measurement may be beneficial to quantify a patient’s inflammation as a therapeutic biomarker.

In our hospital regimen, the oral ganciclovir was considered as more effective than ophthalmic drugs [10]. Thus, we usually give only ganciclovir eye drops when the patients presented mild inflammation and IOP elevation up to 30 mmHg. However, in patients with severe progression of glaucoma, both oral and ophthalmic medication as the first regimen were routinely used. For some who had already used multiple ophthalmic drugs, including steroid and glaucoma eye drops, further eye drops impede treatment adherence. In such cases, only oral medication is used. When we give oral ganciclovir to patients, we take into consideration the renal and hepatic function. What is important is to identify the necessity of trabeculectomy promptly because these patients deteriorate visual field defect rapidly. Therefore, we recommend antiviral therapy as the first regimen but would not hesitate to perform trabeculectomy when this therapy fails. Further studies were needed to investigate the optimal medication for CMV-related uveokeratitis patients.

In conclusion, secondary glaucoma induced by CMV-related uveokeratitis may damage visual field rapidly. Trabeculectomy not only decreases and stabilizes IOP but may contribute to the withdrawal of antiviral medications.

## Data Availability

The data that support the findings of this study are available from the corresponding author, upon reasonable request.
